# Prediction of hypotension during the alveolar recruitment maneuver in spine surgery: a prospective observational study

**DOI:** 10.1186/s40001-023-01031-8

**Published:** 2023-02-03

**Authors:** Ji Young Min, Hyun Jae Chang, Sung Jun Kim, Seung Hee Cha, Joon Pyo Jeon, Chang Jae Kim, Mee Young Chung

**Affiliations:** 1 Department of Anesthesiology and Pain Medicine, Eunpyeong St. Mary’s Hospital, College of Medicine, The Catholic University of Korea, 1021 Tongil-ro, Eunpyeong-gu, Seoul, 03312 Republic of Korea; 2 Department of Anesthesiology and Pain Medicine, Yeouido St. Mary’s Hospital, College of Medicine, The Catholic University of Korea, 10, 63-ro, Yeongdeungpo-gu, Seoul, 07345 Republic of Korea

**Keywords:** Atelectasis, Hypotension, Perfusion index, Recruitment maneuver, Vascular resistance, Vasomotor tone

## Abstract

**Background:**

Atelectasis can occur in many clinical practices. One way to prevent this complication is through the alveolar recruitment maneuver (ARM). However, hemodynamic compromise can accompany ARM. This study aims to predict ARM-induced hypotension using a non-invasive method.

**Methods:**

94 American Society of Anesthesiologists physical status I–II patients aged 19 to 75 with scheduled spinal surgery were enrolled. After anesthesia, we performed a stepwise ARM. Data on perfusion index, mean arterial pressure, heart rate, pleth variability index, cardiac index, and stroke volume variation was collected before induction of anesthesia (T0), just before ARM (T1), at the start of ARM (T2), 0.5 min (T3), 1 min (T4), 1.5 min (T5, end of ARM), and 2 min after the beginning of ARM (T6). Hypotension was defined as when the mean arterial pressure at T5 decreased by 20% or more compared to the baseline. The primary endpoint is that the perfusion index measuring before induction of anesthesia, which reflects the patients’ own vascular tone, was correlated with hypotension during ARM.

**Results:**

Seventy-five patients (79.8%) patients developed hypotension during ARM. The pre-induction persufion index (Pi) (95% confidence interval) was 1.7(1.4–3.1) in the non-hypotension group and 3.4(2.4–3.9) in the hypotension group. (*p* < 0.004) The hypotension group showed considerably higher Pi than the non-hypotension group before induction. The decrease of Pi (%) [IQR] in the non-hypotensive group (52.8% [33.3–74.7]) was more significant than in the hypotensive group. (36% [17.6–53.7]) (*p* < 0.05) The area under the receiver operating characteristic curve of Pi for predicting hypotension during ARM was 0.718 (95% CI 0.615–0.806; *p* = 0.004), and the threshold value of the Pi was 2.4.

**Conclusion:**

A higher perfusion index value measuring before induction of anesthesia can be used to predict the development of hypotension during ARM. Prophylactic management of the following hypotension during ARM could be considered in high baseline Pi patients.

## Background

Lung atelectasis can occur after induction of anesthesia, during surgery, and during mechanical ventilation in the intensive care unit [1]. Atelectasis is common during anesthesia and is often seen in critically ill patients with various underlying etiologies and pathophysiology. This phenomenon can lead to respiratory complications such as pneumonia, hypoxemia, local inflammation, ventilator-induced lung injury (VILI), and increased morbidity [2, 3].

One way to prevent this respiratory complication is through the alveolar recruitment maneuver (ARM). The ARM aims to recover collapsed lungs by steadily increasing transpulmonary pressure until the total vital capacity is reached and may help to prevent impending collapse. In the past few years, it has been hypothesized that ARM in collapsed lungs can protect the lungs because the ventilator-induced lung injury (VILI) is either minimized or disappear in a re-expanded lung state [4]. There are two ARM methods; the bag squeezing and the stepwise maneuver. The stepwise maneuver may reduce the risk of hyperinflation and hypotension by better controlling the airway pressure increase compared to the bag-squeezing maneuver [5–7].

Although ARM is a proven way of preventing atelectasis, it can be accompanied by hypotension [8]. ARM-induced hypotension is known to be closely related to the underlying volume status. In the animal lung injury model, ARM significantly decreased left-ventricular end-diastolic volume and cardiac output at hypovolemia [9]. Anup Das et al. indicated that a higher-than-normal initial cardiac output might protect against the potentially detrimental effects of high intrathoracic pressures associated with ARM on cardiac function [10]. However, to prevent this adverse effect, administering large amounts of fluids rapidly at once in the normovolemic status can develop other harmful consequence.

This study aims to test the non-invasive perfusion index measuring before induction of anesthesia, which reflects the patients’ own vascular tone, to predict the hypotension induced by a protocolized ARM.

## Materials and methods

### Patients population

This study was approved by the Institutional Review Board (IRB) and Hospital Research Ethics Committee (The Catholic University of Korea, Eunpyeong St. Mary’s Hospital IRB; IRB protocol No. PC21OISI0047) and was registered at Clinical Research Information Service (CRIS, https://cris.nih.go.kr, KCT0006168). It was a prospective physiologic study on patients admitted for elective spine surgery. Written informed consent was obtained from each patient before enrollment after approval of IRB. Between June 2021 and February 2022, 94 American Society of Anesthesiologists (ASA) physical status I–II patients aged 19 to 75 with scheduled elective spine surgery were enrolled. Exclusion criteria were patients with increased intracranial pressure, reduced ventricular function (left ventricular ejection fraction < 40%); pre-existing severe vascular disease or cardiac arrhythmia; implanted pacemaker; autonomic nervous system impairment, and unstable vital signs.

### Anesthesia and hemodynamic monitoring

On arrival in the operating room, the patients were monitored with electrocardiography. The peripheral oxygen saturation and blood pressure were also monitored. The rainbow sensor SET™ (Masimo Corp., Irvine, CA, USA) was attached to the index or the middle finger of the dominant arm of each patient for continuous monitoring of the perfusion index (Pi) and pleth variability index (PVI). The patient state index (PSI) was collected using a SedLine^®^ electroencephalograph sensor (Masimo Corp., Irvine, CA, USA) for anesthetic depth. Before anesthesia, the Pi, PVI, blood pressure, and heart rate (HR) as baseline values were measured and recorded. Anesthesia was induced with a bolus of propofol (1.75 mg/kg) and remifentanil 3.0 ng/ml (by effect target-site control using the Minto pharmacokinetic model) and maintained with sevoflurane (0.8–1.0 age-adjusted minimal alveolar concentration in 100% O_2_) to maintain a target PSI of 25–50. Neuromuscular blockade was achieved with 1.2 mg/kg of rocuronium administration. After intubation, sevoflurane was maintained at 0.6 (age-adjusted minimal alveolar concentration) and remifentanil 1.0 ng/ml to minimize the impact of the anesthetic agent on hemodynamics. Mechanical ventilation was performed with an air–oxygen mixture (fraction of inspired oxygen = 0.5) at an 8 ml/kg tidal volume by calculating the ideal body weight. The respiratory rate was 12 breaths per minute to maintain the normocarbia. Positive end-expiratory pressure (PEEP) was not applied. Neuromuscular blockade was monitored through a train of four (TOF) monitoring. The patient’s body temperature was observed through an esophageal thermometer, and the operating room temperature was maintained to ensure that the patient did not develop hypothermia. The A-line was replaced on the radial artery of the side to which the rainbow sensor SET™ was not attached, and mean arterial pressure (MAP), the cardiac index (CI), and stroke volume variation (SVV) were measured using the FloTrac/Vigileo system. The period to stabilize anesthesia from induction of anesthesia to initiate the ARM (This period was designed to abolish any confounding factors of surgical stimulation, patient positioning, and blood loss) was limited to 10 min. An additional 20 mg of esmeron was administered before performing the ARM. There was no stimulation during ARM. An ARM was performed using MAQUET Flow I after switching to pressure mode, lowering driving pressure (Peak pressure—Positive end-expiratory pressure) to 10 cmH_2_O, and adjusting as follows in the supine position:

1. Stepwise increase in end-inspiratory pressure (EIP) up to 35 cmH_2_O.
2. Stepwise increase in positive end-expiratory pressure (PEEP) up to 20 cmH_2_O.
3. Positive end-expiratory pressure (PEEP) after ARM = 5 cmH_2_O.
4. Respiration rate at target = eight breaths per minute (bpm).
5. I: E ratio = 1:1.0.

Pi, PVI, MAP, HR, CI, and SVV were recorded at 0.5-min intervals to monitor hypotension during the alveolar recruitment maneuver. Total ARM time was 1.5 min. The administration of the fluid was restricted to 50 ml/hr. The data were recorded for up to 2 min from the start time of ARM. MAP < 55 mmHg (severe hypotension) was treated by rapid intravenous fluid administration (10 ml/kg) or ephedrine 5 mg bolus if hypotension persisted. Bradycardia was defined as HR < 50 bpm or a decrease by more than 20% below baseline value, whichever was lower, and was treated with atropine 0.5 mg IV boluses. After the completion of ARM, the respiratory rate was adjusted to maintain the normocarbia.

### Data collection

The Pi, PVI, MAP, and HR were collected before induction of anesthesia (T0). The Pi, PVI, MAP, HR, CI, and SVV were collected just before ARM (T1), at the start of ARM (T2), 0.5 min (T3), 1 min (T4), 1.5 min (T5, end of ARM), and 2 min after the beginning of ARM (T6). The MAP at T5 was the lowest; the hypotension group was defined as a case in which the MAP of T5 decreased by more than 20% compared to the baseline.

### Statistical analysis

The number of patients was derived from previously published results. In the previous study, PEEP 5 cmH_2_O was applied, and MAP was each 76 ± 14 mmHg, and 75 ± 11 mmHg in the hemodynamically stable group and the non-hemodynamic stable group [11]. The effect size obtained by G-power was 0.3333333. Ninety-four patients were necessary to achieve a power of 90% with a 5% type I error rate. Data were represented as a number (percentage), median (interquartile range, IQR), or mean (± sd) for quantitative variables after checking for normality. Independent *t*-test or Mann–Whitney *U* test was used to compare continuous variables, and the chi-square test or Fisher's exact test was used to compare categorical variables. A linear mixed model was used to analyze repeated measurements at multiple time points. A *p*-value of < 0.05 was considered significant. If the bivariate correlation achieved statistical significance, logistic regression was performed to determine independent predictability for predicting hypotension. The cut-off value and areas under the receiver operating characteristic (ROC) curves for statistical significance variables were constructed to predict hypotension.

## Results

### Patients’ demographics

**Fig. 1:**
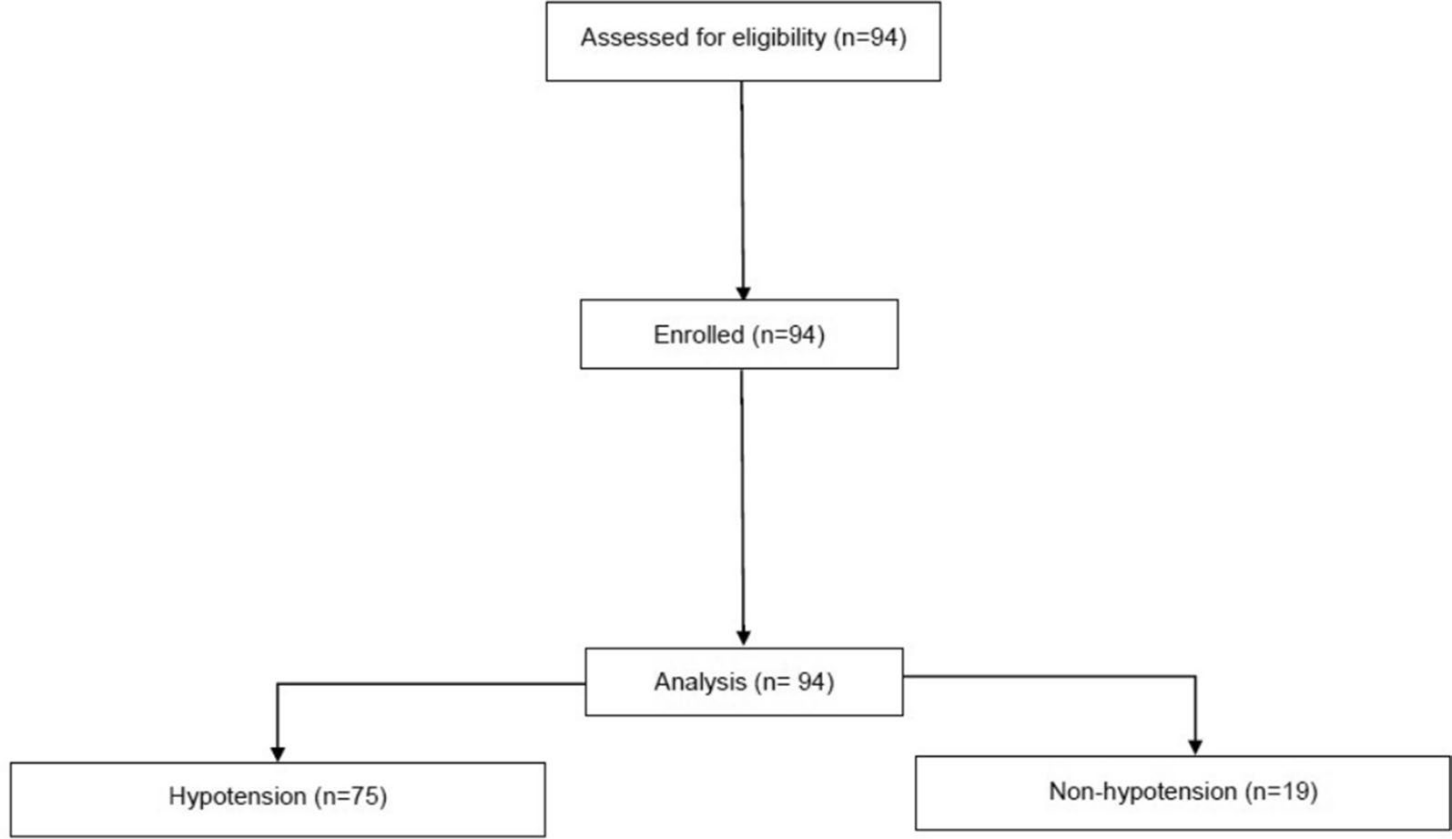
Consort diagram

Of the 94 patients enrolled, 75 patients (79.8%) developed hypotension during ARM (Fig. 1). The lowest MAP occurred in the final stage of ARM (T5) in the hypotension group. Blood pressure quickly recovered from hypotension as soon as the ARM was completed, and its duration was concise. The patients’ characteristics are shown in Table 1, and apart from age, there were no differences between the non-hypotension and hypotension groups.

**Table 1:** Patients’ characteristics

	Non-hypotension (n = 19)	Hypotension (n = 75)	*p* value
Age (years)	50.2 ± 14.7	57.9 ± 10.7	0.01
Sex (male, %)	14 (73.7)	36 (48.0)	0.05
ASA physical status			0.07
I	11 (57.9)	26 (34.7)	
II	8 (42.1)	49 (65.3)	
Comorbidities			0.247
Hypertension	2 (10.5)	20 (26.7)	0.140
Diabetes mellitus	1 (5.3)	7 (9.3)	0.572
Hypertension and diabetes mellitus all	4 (21.1)	12 (16)	0.603
Coronary artery disease	0 (0)	1 (1.3)	0.615
Cerebrovascular disease	0 (0)	2 (2.7)	0.474
Propofol dose (mg)	108.9 (23.0)	118.4(21.2)	0.091
Cumulative remifentanil dose (mcg)	68.8 [27.3–88.6]	74.7 [62.5–89.9]	0.694
Lung compliance (ml/cmH_2_0)	59.4 ± 12.4	52.5 ± 13.1	0.252

Values are presented as mean ± standard deviation, a number of patients (%), or median [interquartile range]

*ASA* American Society of Anesthesiologists

### Hemodynamic variables

**Fig. 2:**
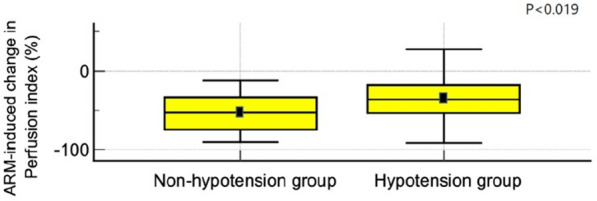
Alveolar recruitment maneuver—induced change in perfusion index (%). The perfusion index difference between T1 (just before ARM) and T5 (end of ARM) showed a significant difference between the two groups. *ARM* alveolar recruitment maneuver

Hemodynamic data are presented in Table 2. Before induction of anesthesia (T0), there was no significant difference in MAP, HR, and PVI between the two groups. (Each *p* = 0.114, 0.292, 0.970) The Pi [interquartile range, IQR] was 1.7 [1.3–2.9] in non-hypotension group and 3.4 [2.3–5.2] in the hypotension group. The hypotension group showed considerably higher Pi than the non-hypotension group before induction. (*p* = 0.004) From 30 s after starting ARM (T3), the MAP (mean ± SD) in the hypotension group was 79 ± 11 mmHg; this result was significantly lower in the hypotensive group than in the non-hypotension group. CI [IQR] was significantly lower in the hypotension group than in the non-hypotension group 1 min after starting ARM (T4) (1.8 [1.4–2.5], 2.3 [2.0–3.1]). SVV [IQR] was significantly higher in the hypotension group than in the non-hypotension group at the end of ARM (T5). (27 [21–33], 18 [17–22]) The Pi was comparable between the two groups during ARM. But there was a significant difference in Pi between the two groups at T5 (end of ARM) when the intrathoracic pressure was maximum. (*p* < 0.05) At this time, Pi was higher in the hypotension group than in the non-hypotension group. The Pi difference between T1 (just before ARM) and T5 (end of ARM) showed a significant difference between the two groups. (Fig. 2) The decrease of Pi (%) [IQR] in the non-hypotension group (52.8% [33.3–74.7]) was more significant than in the hypotension group. (36% [17.6–53.7]).

**Table 2:** Changes in hemodynamic variables

	T0	T1	T2	T3	T4	T5	T6	*p*_Group×Time_
MAP (mmHg)								< 0.001
Non-hypotension	100 ± 9	88 ± 11^†^	90 ± 9	89 ± 10	100 ± 24	101 ± 24	108 ± 18^†^	
Hypotension	105 ± 11	90 ± 11^†^	86 ± 11^†^	79 ± 11^*†^	68 ± 13^*†^	58 ± 15^*†^	74 ± 17^*†^	
HR (beats/min)								< 0.001
Non-hypotension	76 ± 13	83 ± 12	79 ± 11	79 ± 12	89 ± 18^†^	98 ± 21^†^	95 ± 18^†^	
Hypotension	73 ± 12	80 ± 13^†^	76 ± 12	73 ± 11	73 ± 14^*^	73 ± 16^*^	80 ± 15^*†^	
PVI								0.613
Non-hypotension	13 [11–22]	16 [6–20]	14 [8–18]	12 [7–16]	11 [7–15]	13 [10–15]	15 [13–17]	
Hypotension	15 [11–19]	11 [8–18]^†^	11 [7–17]^†^	10 [7–16]^†^	11 [8–17]^†^	13 [10–18]	16 [13–21]	
Pi								0.024
Non-hypotension	1.7 [1.3–2.9]	5.5 [4.5–8.1]^†^	4.9 [3.2–7.3]^†^	5.1 [3.1–7.1]^†^	2.7 [1.6–5.0]	2.5 [1.6–3.4]	3.6 [2.0–4.5]	
Hypotension	3.4 [2.3–5.2]^*^	5.8 [4.1–7.0]^†^	5.3 [3.7–6.7]^†^	5.1 [3.7–6.3]^†^	3.5 [2.1–5.8]	3.3 [2.5–4.4]^*^	3.6 [2.2–4.9]	
SVV								0.009
Non-hypotension		9 [7–12]	9 [8–10]	9 [8–11]	10 [8–15]	18 [17–22]^†^	23 [14–24]^†^	
Hypotension		10 [7–13]	10 [8–13]	11 [8–13]^†^	14 [9–20]^†^	27 [21–33]^*†^	30 [20–35]^*†^	
CI								0.050
Non-hypotension		2.6 [2.1–3.1]	2.8 [2.2–3.3]	2.6 [2.1–3.2]	2.3 [2.0–3.1]	2.1 [1.7–3.1]	2.9 [2.3–3.5]	
Hypotension		2.6 [2.1–3.3]	2.6 [2.0–3.3]	2.5 [1.9–3.0]^†^	1.8 [1.4–2.5]^*†^	1.2 [0.6–1.7]^*†^	2.1 [1.4–2.6]^*†^	

Values are presented as mean ± standard deviation or median [interquartile range]

*T0* induction of anesthesia; *T1* just before ARM *T2* initiation of ARM; *T3* 0.5 min; *T4* 1 min; *T5* 1.5 min; *T6* 2 min after initiation of ARM; *MAP* mean arterial pressure; *HR* heart rate; *PVI* pleth variability index; *Pi* perfusion index; *SVV* stroke volume variation; *CI* cardiac index

*p*_Group×Time_, *p*-value of the group, and time interaction obtained by linear mixed model analysis

^*^p < 0.05 compared with the non-hypotension group

^†^*p* < 0.05 compared with the value of “T0” in each group in MAP, HR, PVI, and Pi

^†^*p* < 0.05 compared with the value of “T1” in each group in CI, SVV

### Prediction of hypotension

**Fig. 3:**
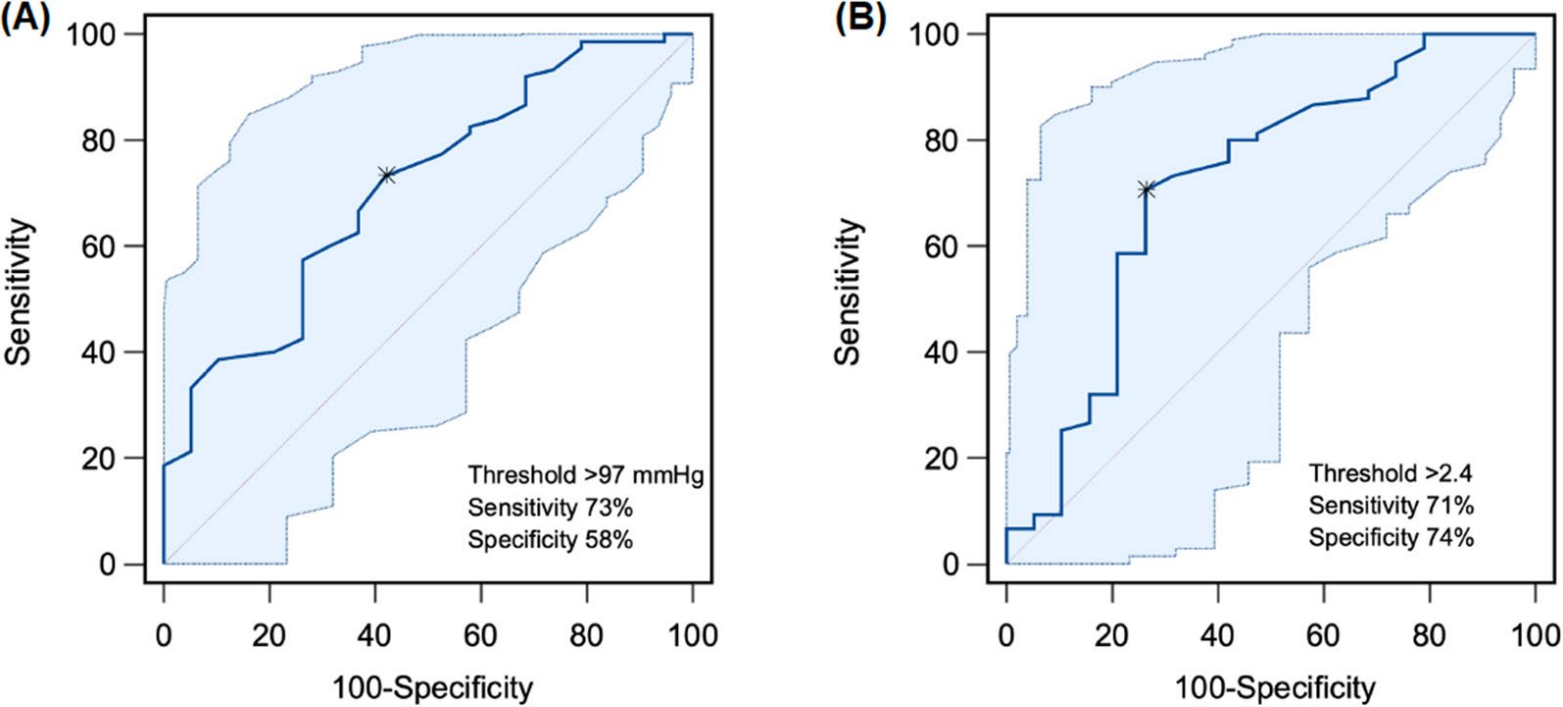
Areas under the receiver operating characteristics curves (AUCs) of each index measuring before induction of anesthesia for predicting hypotension during alveolar recruitment maneuver (ARM). **A** MAP, **B** Pi. *CI* confidence interval; *MAP* mean blood pressure; *Pi* perfusion index

Areas under the receiver operating characteristic curves (AUCs) of each index measuring before induction of anesthesia for predicting hypotension during ARM are presented in Table 3. Among the variables, the Pi (AUC 0.718; 95% confidence interval 0.615–0.806; *p* = 0.004) showed AUC > 0.7. The optimal threshold value of the Pi was 2.4 (sensitivity 71%, specificity 74%) (Fig. 3).

**Table 3:** The area under the receiver operating characteristic curves of each index measuring before induction of anesthesia for prediction of ARM-induced hypotension

	The area under the curve	95% confidence interval	*p* value
MAP (mmHg)	0.710	0.607–0.799	0.001
HR (beats/min)	0.593	0.487–0.694	0.230
PVI	0.503	0.398–0.608	0.971
Pi	0.718	0.615–0.806	0.004

*ARM* alveolar recruitment maneuver*; MAP* mean arterial pressure; *HR* heart rate; *PVI* pleth variability index, *Pi* perfusion index

## Discussion

This prospective observational study investigated the hemodynamic variables for predicting hypotension during the ARM. We found that Pi > 2.4 in the pre-induction period can be used to predict hypotension during ARM with reliable sensitivity and specificity.

In the study of fluid responsiveness, PVI and SVV was valuable parameter to predict fluid responsiveness in the operating room. In hypovolemia, the left ventricle usually functions on the ascending part of the Frank–Starling curve [12–16]. Thus, PVI and SVV should be more distinct when compared with that normovolemic state. If SVV or PVI shows a higher value, it may be indicated a low volume status. In the present study, pre-induction PVI was no significant difference between the two groups. After induction of anesthesia, PVI, CI, and SVV also showed no significant difference between the two groups. As these parameters were no significant difference in the pre-induction and post-induction period before ARM in the present study, we assumed that the preload of the patients was similar. Furthermore, the value of PVI, CI, and SVV were also within the normal range, the patient was assumed to be in a normovolemic status. In addition, the lung compliance of the hypotension and non-hypotension groups showed no significant difference. Therefore, the interaction between the heart and lungs was also assumed to be similar in the two groups.

Theoretically, hypotension during ARM is caused by a reduced preload secondary to a decrease in venous return generated by increased intrathoracic pressure that compresses the heart. In a previous study, the preload decreases measured echocardiographically were correlated with transient hypotension during the ARM while the contractility was maintained [17]. When mechanical pressure of the ARM generates, the short-term mechanism for compensation would be increasing the vascular tone. A vascular tone rises due to the activation of a sympathetic tone. In the experimental animal study, the vascular tone was increased, mediated by the carotid baroreceptor reflexes, to compensate for the hemodynamic inhibition of PEEP. When the effect of PEEP exceeds this compensatory mechanism, hypotension occurs [18]. Pi, measured from a plethysmograph signal of the pulse oximeter, is calculated as the ratio between the pulsatile component (arterial compartment) and the non-pulsatile component (venous and capillary blood and other tissues) of the light reaching the detector of the pulse oximeter, able to monitor vascular tone and correlate with vascular resistance [19, 20]. An alteration of the Pi is mainly affected by changes in the pulsatile component [20]. When the pulsatile component is declining by vasoconstriction (higher SVR), low Pi appears, and when the pulsatile component is enhanced by vasodilation (lower SVR), high pi appears [21].

In the present study, the hypotension group had a high baseline Pi than the non-hypotension group. When high intrathoracic pressure caused by ARM was applied to patients with similar cardiopulmonary interactions and volume status, patients with a low vascular tone, i.e., low SVR, did not tolerate it well. In other words, ARM-induced hemodynamic suppression was more significant in patients with already high Pi, who have lower basal vascular resistance. Suppose the hemodynamic inhibition of ARM was applied in the already vasodilated state. In that case, continuous compensatory vasoconstriction, Blevins et al. [18] mentioned in their study, may not have occurred in patients with already baseline vascular resistance may be down.

Blood pressure was well maintained in the non-hypotension group since the degree of compensation was more significant in the non-hypotension group. The decrease in Pi was 52.8% [33.3–74.7] in the non-hypotension group and 36% [17.6–53.7]) in the hypotension group. The decrease between Pi just before ARM (T1) and at the end of the ARM (T5) in the non-hypotension group was more significant than in the hypotension group. This means that Pi decreased more, suggesting vasoconstriction may be sufficient to overcome ARM-induced hypotension in the non-hypotension group. The non-hypotension group maintained some degree of compensatory vasoconstriction by increasing vascular tone when the hemodynamic decline occurred. On the other side, in the hypotension group, the already lowered vascular tone did not defend well against the inhibitory effect of ARM.

Unlike CI and SVV, the PVI did not change during ARM in the present study. The degree of change in Pi according to the respiration cycle is PVI, no significant difference in PVI in the present study may mean that Pi did not change much with the respiratory cycle. The adverse effect of ARM provides a strong stimulus to the sympathetic system; this leads to vasoconstriction, that is, a decrease in Pi. In other words, if sympathetic nerve activation must continue to occur to compensate for the adverse effect of ARM, changes according to the respiratory cycle may have less influence than conventional mechanical ventilation. Under conditions in which the Pi changes substantially in response to the robust stimulus of ARM, the PVI may not be calculated from the respiratory variations in the Pi but rather from the variation in the Pi induced by the fluctuation in vasomotor tone [22]. In case vasoconstriction was continuously required to maintain blood pressure, Pi, which represents vascular tone, may be likely to remain low. Unlike the usual mechanical ventilation of repeated inspiration and exhalation, stepwise ARM has a plateau period in which high positive pressure is maintained instead of exhalation time, so this different respiratory cycle may also have influenced the change in PVI. Moreover, the FloTrac/Vigileo system, which calculates the stroke volume based on an arterial pulse contour analysis, is influenced by factors that affect the arterial blood pressure and the arterial pressure waveform independently of the stroke volume. The SVV might be less affected than PVI by changes in the vasomotor tone.

Desebbe et al. [11] found that PVI can be helpful in noninvasively predicting the hemodynamic effects of PEEP. In their study, PVI can predict the hemodynamic effects of 10 cm H_2_O of PEEP in mechanically ventilated patients after cardiac surgery. The present study included relatively healthy patients, but previous studies included cardiac surgery patients with mechanical ventilation. Patients undergoing cardiac surgery may have significantly different hemodynamic statuses than other groups. Therefore, the application of Pi must be correlated with the clinical status of the patients.

Although ARM was performed once in the present study, a periodic repetition of ARM may reduce respiratory complications in many clinical practices, including intensive care units [23, 24]. Therefore, the incidence of hypotension may be higher than in the present study, and repeated exposure to such hypotension may lead to impaired organ perfusion. Many studies have demonstrated that frequent exposure to hypotension in the intraoperative period affects postoperative cardiac, renal, or neurological adverse events [25–27]. Based on the results of the present study, it can be suggested that if the baseline Pi is 2.4 or higher before ARM, it may be helpful to reduce exposure to hypotension by prophylactic pretreatment. Also, most critically ill patients in the intensive care unit are vasodilated with co-morbidity. Therefore, it is necessary to pay close attention when implementing ARM. It may be possible to reduce side effects by observing Pi in these patients.

Our study has several limitations. First, most medications can be taken on the day of surgery; preoperative hemodynamic status might have been affected if the patient took circulatory medications, such as calcium channel blocker (CCB), Angiotensin receptor block (ARB), or angiotensin-converting enzyme inhibitors (ACEi). CCB was taken on the day of surgery, but neither ACEi nor ARB was taken on day of surgery. Therefore, it is possible to reduce the benefit observed on the Pi. Second, most of the patients included in the analysis were elderly; older people are more susceptible to anesthetics due to physiological changes from aging or co-morbidity. Moreover, the elderly have reduced physiological responses to hemodynamic changes. Third, we used a relative threshold for defining hypotension. The present study described hypotension as a case in which the MAP of T5 decreased by more than 20% compared to the baseline. Because Pi is the by-product of vascular tone, defining hypotension as a function of baseline MAP is probably more physiological. Also, measuring pre-morbid basal blood pressure and customizing it according to the clinical response may be needed in patients [28–30].

## Conclusion

In conclusion, the Pi measuring before induction of anesthesia could be an indicator for predicting ARM-induced hypotension. If the patient has a Pi > 2.4 before the induction of anesthesia, prophylactic management of the following hypotension during ARM could be considered. However, the application of Pi must be correlated with the clinical status of the patients.

## Data Availability

The datasets used and analyzed during the current study are available from the corresponding author upon reasonable request.

## Abbreviations

AUC: Area under the curve
ARM: Alveolar recruitment maneuver
CI: Confidence interval
HR: Heart rate
MAP: Mean arterial pressure
MBP: Mean blood pressure
PVI: Pleth variability index
Pi: Perfusion index
SpO_2_: Oxygen saturation
ROC: Receiver operating characteristic
